# Bridging the gap between clinicians and systems biologists: from network biology to translational biomedical research

**DOI:** 10.1186/s12967-016-1078-3

**Published:** 2016-11-22

**Authors:** Natini Jinawath, Sacarin Bunbanjerdsuk, Maneerat Chayanupatkul, Nuttapong Ngamphaiboon, Nithi Asavapanumas, Jisnuson Svasti, Varodom Charoensawan

**Affiliations:** 1 Integrative Computational BioScience (ICBS) Center, Mahidol University, Nakhon Pathom, Thailand; 2 Program in Translational Medicine, Faculty of Medicine Ramathibodi Hospital, Mahidol University, Bangkok, Thailand; 3 Department of Physiology, Faculty of Medicine, Chulalongkorn University, Bangkok, Thailand; 4 Division of Gastroenterology and Hepatology, Department of Medicine, Baylor College of Medicine, Houston, TX USA; 5 Medical Oncology Unit, Department of Medicine Faculty of Medicine, Ramathibodi Hospital, Mahidol University, Bangkok, Thailand; 6 Department of Physiology, Faculty of Science, Mahidol University, Bangkok, Thailand; 7 Department of Biochemistry, Faculty of Science, Mahidol University, Bangkok, Thailand; 8 Laboratory of Biochemistry, Chulabhorn Research Institute, Bangkok, Thailand; 9 Systems Biology of Diseases Research Unit, Faculty of Science, Mahidol University, Bangkok, Thailand

**Keywords:** Network biology, Systems biology, Biomedical research, Cancers, Personalized therapy

## Abstract

With the wealth of data accumulated from completely sequenced genomes and other high-throughput experiments, global studies of biological systems, by simultaneously investigating multiple biological entities (e.g. genes, transcripts, proteins), has become a routine. Network representation is frequently used to capture the presence of these molecules as well as their relationship. Network biology has been widely used in molecular biology and genetics, where several network properties have been shown to be functionally important. Here, we discuss how such methodology can be useful to translational biomedical research, where scientists traditionally focus on one or a small set of genes, diseases, and drug candidates at any one time. We first give an overview of network representation frequently used in biology: what nodes and edges represent, and review its application in preclinical research to date. Using cancer as an example, we review how network biology can facilitate system-wide approaches to identify targeted small molecule inhibitors. These types of inhibitors have the potential to be more specific, resulting in high efficacy treatments with less side effects, compared to the conventional treatments such as chemotherapy. Global analysis may provide better insight into the overall picture of human diseases, as well as identify previously overlooked problems, leading to rapid advances in medicine. From the clinicians’ point of view, it is necessary to bridge the gap between theoretical network biology and practical biomedical research, in order to improve the diagnosis, prevention, and treatment of the world’s major diseases.

## Background

Next-generation sequencing (NGS) and other high-throughput experiments highlight one of the most significant advances in molecular biology over the past decade. Such technological improvements enable a large number of molecules, including genes, transcripts, and proteins to be simultaneously measured in different conditions over time. This rapid generation of data has transformed molecular biology from a “data poor” to “data rich” discipline, leading to the emergence of systems biology [1–4]. The key challenges and bottlenecks of the modern-day molecular biology have shifted from simply gathering information to the analysis and interpretation of large quantities of data that can now be obtained.

Network representations have been widely used in physics and social science for decades, and are now among the most frequently used tools in systems biology. This technique provides not only a systematic representation of both the presence and abundance of biological molecules, but also displays the relationships or interactions between them. Networks have been used to represent the interactions between different types of biological molecules, e.g. protein–protein interactions [5–8], and in various biological systems including transcriptional regulation [9–11], signaling [12–14], and metabolic pathways [15, 16]. Analyses of network sub-structures have revealed fundamental insights into how biological molecules are organized [17–20], which would not have been possible by studying individual genes or proteins.

Network representation and analysis has been successfully applied to study many systems in molecular biology [21]; however, the use of these tools in translational medicine and drug discovery is relatively new [22–24]. This might be due in part to the knowledge and understanding gaps between clinicians and systems biologists. By convention, clinicians typically focus on specific sets of key genetic markers associated with diseases, to identify the most probable drug targets. In contrast, systems biologists have strong computational and analytical skills, but frequently lack hands-on experimental experience. The lack of interaction of systems biologists with patients can prevent a full appreciation of the complexity of the problems and hindrances in biomedical research [25, 26]. In this review, we aim to improve the understanding of challenges in biomedical research and establish a common ground between clinicians and systems biologists to further promote the application of network biology in translational medicine.

## Network biology in a nutshell

### What are networks; what do they represent?

**Fig. 1:**
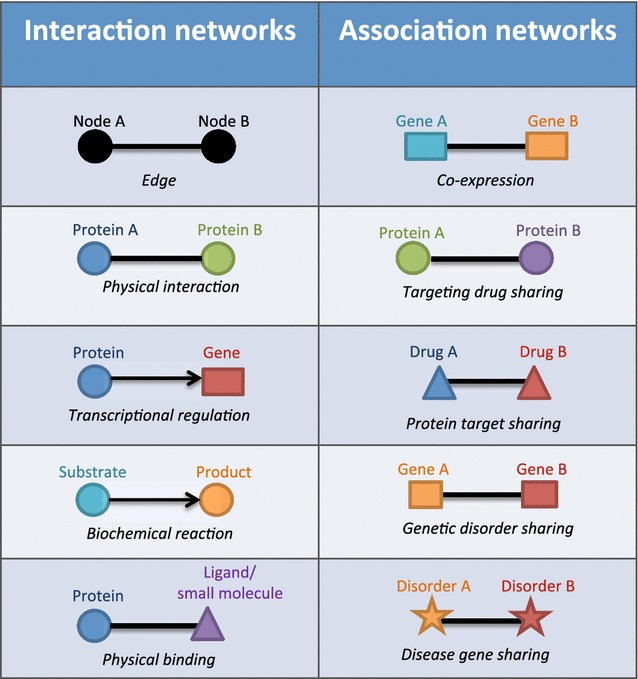
Interaction networks (*Left*) represent direct interactions between biological molecules (e.g. transcripts, proteins, and ligands). The interactions represented include direct physical interaction (e.g. protein–protein, and gene regulatory networks) or transition (e.g. metabolic network). Association networks (*Right*) represent biological molecules that are linked based on their shared and/or common properties (e.g. co-expression)

We first outline the fundamental concepts of a network representation. In general, a network represents the presence of objects or entities in a system as “nodes”, and the relationships or interactions among the nodes are called “edges” (Fig. 1). In biology, nodes can represent biological molecules such as genes, proteins, and ligands, or even larger entities such as cells or individual humans. Edges represent physical interactions or contacts between biological molecules, biochemical processes between substrates and products, genetic interactions between genes, and in some cases, interactions between cells or individual organisms.

Biological information described in a network is not restricted to the presence of nodes and their relationships. The size of node, for instance, can reflect abundance of biological molecules (e.g. gene expression levels). Nodes can also be drawn in different shapes and/or colors according to the classification of interest (e.g. gene/protein family). Likewise, the thickness of an edge or the distance between nodes may represent the frequency or strength of pairwise interaction (e.g. affinity of protein–protein interaction); whereas colors can indicate different types of interactions (e.g. physical or genetic interaction). In addition, edges can be directional or non-directional, solid or dotted, depending on the types of interactions. Thus, networks are information-rich representations, which are widely used to summarize, visualize, and analyze large-scale datasets obtained from high-throughput experiments. To give an overview of the current application of networks in biomedical-related fields, here we review two major types of biological networks.

### Interaction networks

We first illustrate the components of interaction networks, where the edges represent a “direct” relationship between nodes (Fig. 1, left). For instance, protein interaction networks, i.e. interactomes, describe physical interactions between proteins, usually obtained from high-throughput screening techniques such as yeast-two hybrid [6, 27], or affinity purification followed by mass spectrometry [5, 28]. In humans, analyses of protein–protein interaction networks have shown that dysfunctional interactions can lead to several diseases including neurological disorders such as ataxias [29], autism [30], several types of cancers including breast [31] and colorectal cancers [32], acute lymphoblastic leukemia [33], as well as other inheritable genetic diseases [34–37].

Transcriptional regulation networks (also known as Gene Regulatory Networks, GRNs) are widely used to illustrate the binding events of regulatory proteins, such as transcription factors, to the promoters of targeted genes, and this technique has been employed in the analysis of bacteria [38], budding yeasts [9], worms [39], and embryonic stem cells [40, 41]. GRNs are directional, and the relationship between two nodes is represented by an arrow starting from a regulator and pointing toward a targeted gene. Mis-regulation of gene expression leads to various diseases especially cancers, as seen in the genome-wide transcription network of the vertebrate transcription factor SOX4 [42], and the androgen receptor, a transcription factor that regulates the onset and progression of prostate cancer [43].

Interaction networks have also been used to describe the binding and affinity of ligands or small molecules to targeted proteins. As seen in a drug-target network [44], a list of drugs approved by the Food and Drug Administration (FDA) were linked to proteins according to drug-target binary associations. The analysis of these networks revealed that many drugs have overlapping but not identical sets of targets. In addition, the network analysis indicated that new drugs tend to be, at least partly, linked to well-characterized proteins already targeted by previously developed drugs. This suggests that the pharmaceutical industry might be shifting toward polypharmacology, to systematically address complex diseases using multiple drugs aimed at multiple specific targets in related pathways to improve treatment efficacy [45, 46].

Metabolic networks differs from other networks described earlier in the sense that the edges between two nodes (metabolites) do not represent physical contacts, but instead biochemical reactions that convert one metabolite to another. Recent studies have reconstructed and explored genome-scale metabolic networks in pathogenic microbes including *Staphylococcus aureus* [47], *M. tuberculosis* [48], as well as in human hosts [49]. These analyses may lead to a better understanding of host-pathogen interactions, and could aid in the design of drugs that specifically target the metabolic pathways of microbes and cause minimal interference with those of the hosts.

### Association networks

Networks can also be used to visualize and summarize the overlap in expression profiles for thousands of transcripts/proteins obtained from high-throughput methods, such as expression microarray, RNA-seq, or short-gun proteomics [50]. In co-expression networks, two or more genes are linked if their products (mRNAs or proteins) exhibit similar expression profiles, with the strength/thickness of the edges proportional to how often the two transcripts are expressed at the same time and/or place [51, 52]. Co-expression networks are widely used as a starting point for inferring the cellular functions of uncharacterized genes, as in many cases, genes with related functions show overlapping expression patterns [53]. New disease markers can be discovered from clusters of genes that are co-expressed with known disease-associated genes, as they frequently show differential expression between the normal and diseased populations [54–57].

Other association networks include drug target-protein networks [44], where each node is a protein and two proteins are linked if they are targeted by the same compounds. These networks can be computationally derived from the drug-target network described in the previous section. It provides a complementary protein-centric view by focusing on the proteins that are often co-targeted, and might be involved in related pathways. Conversely, two or more drugs can be linked in a network based on common properties, such as targeting specific proteins or side effects. It has been shown that documented adverse side effects could be used to infer molecular drug-target interactions [58]. This type of network has the potential to predict whether or not existing and routinely used drugs have additional unknown off-targets, allowing for these drugs to be candidates for additional, distinct therapeutic categories. Illustrations of the potential of alternative uses for current drugs are sildenafil, losartan, and fenofibrate. Sildenafil (e.g. Viagra^®^, Pfizer Incorporated) was initially developed to treat angina, but a side effect (prolong penile erection) discovered during clinical trial has become its main use. The antihypertensive drug losartan blocks angiotensin II type 1, and is now a candidate drug for preventing aortic aneurysm complications in Marfan syndrome patients, through reduction of TGF-β activitiy [59, 60]. Fenofibrate, a drug mainly used for controlling cholesterol levels in cardiovascular patients, has also been shown to suppress growth of hepatocellular carcinoma [61].

Global disease networks offer a useful insight into how human disorders are related. In the “human disease network” [62], disease nodes are connected if they share at least one gene with mutations associated with both diseases. Complementarily, the gene-centric version of this network comprises nodes of disease genes, linked if they are associated with the same disorders. Such networks not only represent a framework to visualize all known disease genotype-phenotype associations, but also reveal that human diseases are much more genetically related than previously appreciated [63]. This is highlighted by a gigantic network comprising over 500 interconnected human diseases [7].

### What can we learn from networks and their properties?

In addition to being a framework for visualizing and documenting all the known relationships between nodes, earlier analyses of large-scale networks from high-throughput studies have revealed many interesting biologically relevant properties, which cannot be obtained by studying genes and proteins individually [64–66]. One of the most frequently observed properties of biological networks is the connectivity distribution that follows a power-law distribution, known as “scale-free networks”. This pattern of connections, also known as the “small world property”, has also been extensively studied for their statistical features in different types of networks, including social networks, scientific collaboration networks, and the World Wide Web [67–72]. In brief, a scale-free network consists of a small number of “hubs”, i.e. nodes that are connected to a larger number of other nodes, through different types of interactions aforementioned. In contrast to hubs, the majority of nodes in the network have much fewer connections. Several studies have documented similar observation for biological networks, including protein–protein interaction networks [6, 17, 73] and metabolic networks [15, 74].

Because of their connectivity distribution, scale-free networks are robust against random deletion of nodes. That is, the connections between a node and most other nodes remain intact, if nodes are removed randomly. In contrast, scale-free networks quickly become non-functional if hubs are targeted. Earlier studies have shown that many pathogenic organisms have evolved to target the central components (i.e. hubs) of a human protein interaction network, and quickly disrupt various cellular functions, including the immune response [75, 76]. Similarly, one would expect drugs that specifically inhibit the central components of the regulatory circuits in a pathogen will rapidly disrupt their homeostatic processes, and thus efficiently eliminate them. As a result, these hubs from pathogenic organisms could be promising candidates for novel drugs. Network connectivity distribution is one of the better-studied areas, and a number of insightful reviews and analyses are available [77, 78].

Another interesting example of biological network properties are the network motifs, which are sets of well-defined interconnection patterns between nodes [19]. These connectivity patterns, or network sub-circuits, recur in biological networks at a frequency significantly higher than in randomized networks [79–81], signifying their important roles as building blocks for the large-scale organization of interactions. The patterns and proportions of sub-circuits used in different networks are distinct, depending on the functionality required under different conditions. Interestingly, it has been shown in a yeast transcription regulatory network that sub-network structures, facilitating fast signal propagation (e.g. single-inputs), are more frequently employed to respond to external stressors and sudden environmental changes (e.g. DNA damage or diauxic shift), because a rapid response is required against the stressors. In contrast, motifs that buffer spurious inputs or only respond to persistent signals (e.g. feed-forward-loops) are more suitable for analysis of normal growth stages (e.g. sporulation) [18, 82].

## Applications of network biology in translational medicine

### Disease network and drug discovery

Using a transistor radio as an analog of a biological system, Yuri Lazebnik described how a biologist would fix a broken radio, assuming no prior knowledge of how the radio components were wired together [83]. A traditional biological approach would involve removing (gene knockout, mutagenesis) each part of a functioning radio and track the changes in performance (phenotype). However, the human “radios” are different and repeating this process on all the components would generate an enormous amount of data, some of which may be redundant or contradictory. In contrast, a typical engineering approach would involve systematic reconstruction of a component diagram from a normal radio (e.g. regulatory network), and compare the broken radios with the normal reference. Can a similar problem-solving mindset help expedite advances in biomedical research?

**Fig. 2:**
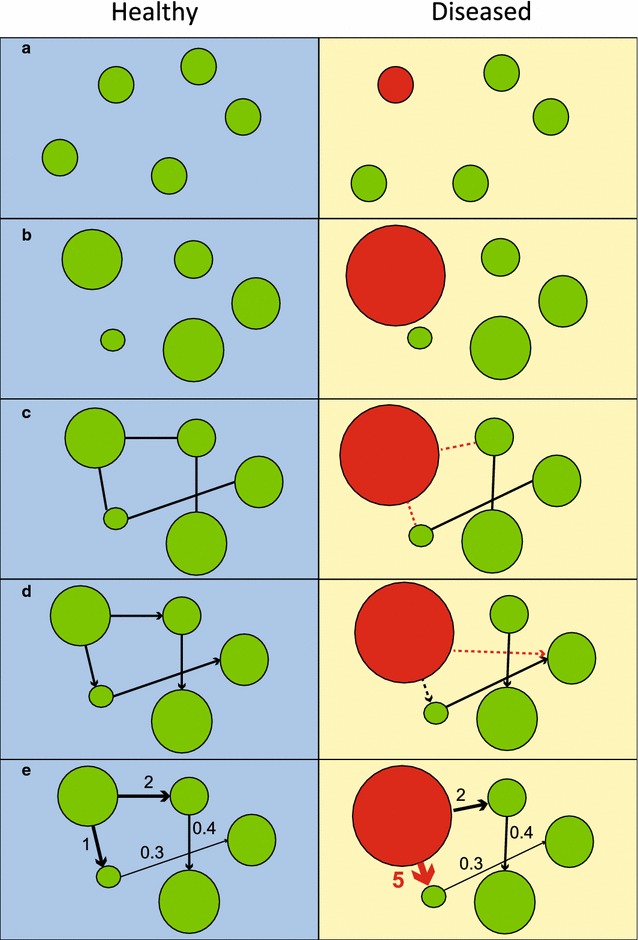
Biological networks of healthy (*left panel*) and diseased (*right panel*) individuals. Biological components in healthy individuals are represented as *green nodes* in a network. Pathological perturbation, represented by *red nodes* that lead to morbidity, can occur at different stages of the regulation of key components: **a** presence and absence of key component (*green* for presence and *red* for absence), **b** mis-regulated gene expression, leading to over- or under-expression (*node sizes* represent expression levels), **c** absence or erroneous interactions with interacting partners (*dotted lines* represent erroneous interactions), **d** mis-regulated directions (*mis-directed arrows*), or **e** strengths of interactions (*thicknesses of arrows* and *accompanying numbers* denote interaction strengths)

If regulatory circuits that control biological activities in a human body can be represented using a complex network, then a diseased state would be expected to occur when the normal state of the network is perturbed. Failure of key components (e.g. mutations in hub genes in genetic diseases) or external stimuli (e.g. invasion of pathogens in infectious diseases) would lead to loss of network integrity. Diseased perturbations can occur at different regulatory levels, as illustrated in Fig. 2. Firstly, the absence or malfunction in important network components can lead to diseases, such as the loss of a particular gene. The absence of *TBX1*, in 22q11.2 deletion syndrome (DiGeorge syndrome) is responsible for the majority of characteristic features of this disease [84] (Fig. 2a, the absence of node is illustrated in red). Similarly, inappropriate levels of gene expression can cause disorders (Fig. 2b, altered node size). For example, specific mutations in the *FGFR3* gene result in an overactive receptor and lead to the short stature phenotype observed in achondroplasia [85]. Some diseased states can be explained by mis-regulation of the interactions between key components of the network (Fig. 2c, missing edge), as well as mis-direction (Fig. 2d, mis-directed edge) or strength (Fig. 2e, altered edge’s thickness) of interactions. The diseases that can be linked to erroneous interactions include neurodegenerative and neurodevelopmental diseases, genetic disorders, and cancers. In these cases, mutations in multiple relevant genes lead to abnormal protein interactions, and disrupt networks (see [29, 30, 36, 37] for details).

Some of the long-standing challenges in drug discovery are lack of specificity, high incidence of adverse effects, and unpredicted toxicities of new therapeutic compounds [86]. As a result, modern-day drug discovery employs more targeted approaches, such as virtual screening and structure-based drug design to complement conventional in vitro high-throughput screening [46, 87]. These new approaches rely on an accurate global understanding of the mechanisms of diseases. Comprehensive understanding of the network and regulatory circuit for a particular disease process would help to identify network hubs with the potential to be novel drug targets.

### A network model of cancers

In the past decades, chemotherapy had been the backbone for systemic treatment of cancers. When administered to patients, these drugs target rapidly dividing cells but lack specificity. Survival of both cancer cells and normal, rapidly growing cells are impaired, resulting in side effects such as bone marrow suppression and hair loss, due to toxicity toward bone marrow cells and hair follicles, respectively. With recent advances in molecular biology and genetics, several genetic mutations and other alterations have been described for various cancers, and these changes specific to cancer cells have become an attractive target for novel therapies. The concept of “driver” and “passenger” mutations in carcinogenesis is comparable to hubs and peripheral nodes in a network, whereby a subset of somatic alterations present in each tumor is a driver of the oncogenic process [88]. Acting as a complex network hub, these driver mutations promote cancer cell survival, resistance to apoptosis, and lead to carcinogenesis (so-called “oncogene addiction”). This idea is supported by successful identification of new cancer fusion drivers from the network hubs and their partners, as the fusion mutation can lead to functional de-regulation of multiple genes and pathways [89]. Inhibition of the driver mutation has the potential to induce cell death, and thus becomes a strong candidate for targeted therapy [90]. As cancer cells are addicted to this driver mutation, specifically blocking these hubs would theoretically be more effective and less toxic compared to conventional chemotherapy.

To date, many targeted therapies have been approved as a standard of care in various cancers with additional clinical studies underway. Identification of a true driver; however, remains one of the biggest challenges. Pathogenesis of cancer development is usually complex and involves several molecules and pathways. Therefore, targeting one particular molecule or pathway might not be effective, as cancer cells may utilize alternative pathways to promote cell survival. Additionally, with the advent of next-generation sequencing, the previously well-accepted but unproven concept of tumor genetic heterogeneity has been solidly confirmed [91]. Sequential use of more than one targeted cancer therapy to finish off resistant clones, such as in the case of tumor recurrence, is likely to become a trend in cancer genomic medicine [92].

### Breast cancer network: mechanisms of resistance

The regulatory network in breast cancer is a particularly interesting case study, due to its heterogeneous histological and molecular features, and clinical manifestations that lead to multiple molecular sub-types. Based on gene expression profiling, breast cancer can be categorized into four main molecular sub-types: (i) basal-like breast cancer (mainly estrogen-receptor (ER)-negative, progesterone-receptor (PR)-negative, and human epidermal growth factor receptor 2 (HER2)-negative); (ii) luminal-A cancer (ER-positive or ER+, and histologically low-grade); (iii) luminal-B cancer (ER+ and histologically high-grade); and (iv) HER2-positive (HER2+) cancer (over-expression and/or amplification of HER2). Each molecular sub-type has a distinct course of disease progression and responds differently to specific treatments, including endocrine therapy, anti-HER2 drugs and cytotoxic chemotherapy [93].

**Fig. 3:**
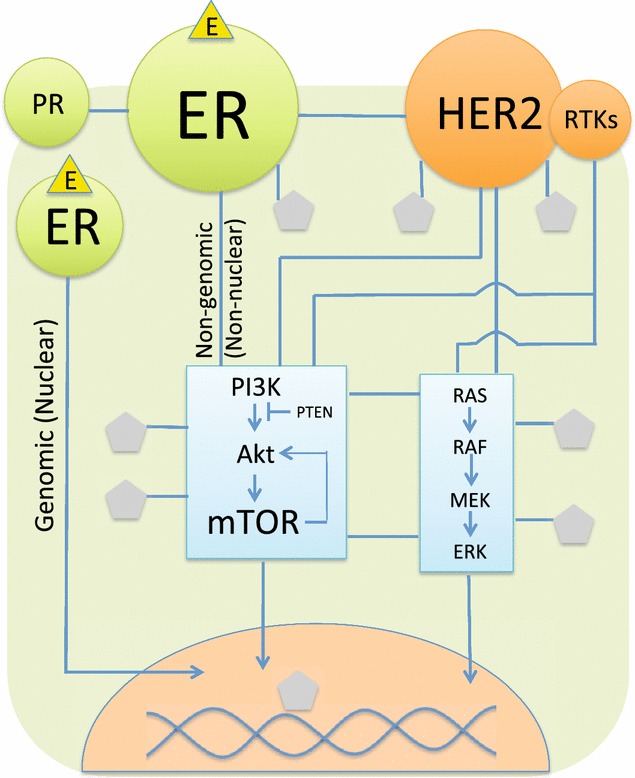
A simplified diagram of the therapeutic breast cancer network. The main targetable hubs are ER and HER2 receptor. The PI3K/Akt/mTOR hub was relatively recently identified to be the common mechanism of targeted therapy resistance. *Circles* and *rectangles* represent cellular receptors and signaling pathways, respectively. The *pentagons* represent other unspecified molecules interacted with the hubs. *Arrows* represent the directions of signals. (*E* estrogen, *ER* estrogen receptor, *PR* progesterone receptor, *HER2* HER2 receptor, *RTKs* receptor tyrosine kinases)

As shown in Fig. 3, ER and HER2 can be considered as hubs of the breast cancer network. The ER+ breast cancer cells depend on activation of ER by estrogen, a sex steroid hormone. ER acts as a transcription factor in the nucleus when bound by estrogen in the genomic (nuclear) pathway, resulting in tumor cell proliferation [94]. The signal can also be activated through the non-genomic (non-nuclear) pathway, where estrogen binds to membrane-associated ER. Endocrine therapy against the ER hubs is one of the cornerstones of treatment for ER+/HER2- breast cancers (luminal-A and B) [95]. The predominant endocrine therapies are a selective ER modulator (SERM), an aromatase inhibitor (AI), and selective ER down-regulators (SERD), such as tamoxifen, anastrozole, and fulvestrant [96].

HER2, a member of the epidermal growth factor receptor tyrosine kinase family, is a hub in the HER2+ breast cancer network. Over-expressed and/or amplified *HER2* is found in approximately 20–30% of invasive breast cancers [97]. HER2 activates intracellular signaling cascades, leading to tumor cell proliferation. Inhibition of HER2 through the use of anti-HER2 drugs significantly prolongs survival in HER2+ breast cancer patients. Currently, several anti-HER2 drugs are FDA-approved for HER2+ breast cancer, including trastuzumab, lapatinib, pertuzumab, and trastuzumab emtansine (T-DM1). Resistance to each of these specific treatments has been observed, as well as interactions between the ER and HER2 hubs (Fig. 3) [94, 98]. Since ER+/HER2+ tumor cells depend on both hubs, endocrine therapy alone cannot completely inhibit signals with tumor cell proliferation continuing to be activated through HER2 (so-called “cross-talk”). This has been identified as a primary mechanism of resistance in ER+/HER2+ breast cancer patients with a low response to endocrine therapy. With a better understanding of global gene regulation networks and the interplay between the two hubs, a combined treatment of endocrine therapy and anti-HER2 drugs was proposed. Several phase 3 clinical studies have already demonstrated increased efficacy of endocrine therapy in the ER+/HER2+ breast cancer when combined with anti-HER2 drugs [99–101].

On the other hand, ER+/HER− breast cancer does not depend on the HER2 hub, and is thus usually responsive to the first line endocrine therapy. However, resistance can still occur leading to less effective endocrine therapy. Blocking the ER hub with any endocrine therapy would inhibit only the genomic pathway, but not the non-genomic pathway where abnormal activation of the PI3K/Akt/mTOR pathway by somatic mutations can result in either *de novo* or acquired endocrine therapy resistance [102, 103]. Understanding this relationship has led to a second line of endocrine therapy using mTOR inhibitors. A large phase 3 clinical study of metastatic ER+/HER2− breast cancer patients, who failed the first line AI treatment, reported longer progression-free survival in a group treated with a combination of an mTOR inhibitor and another different AI [104, 105].

Having a comprehensive understanding of the interactions between network components of specific disease should lead to improved efficacy in treatments, similar to those elucidated using the breast cancer model above. Indeed, a number of groups have already begun utilizing network biology to address different aspects of cancers with the goal to improve diagnosis and treatment. A model to identify genes potentially associated with high risks of breast cancer has been developed by integrating data from co-expression, biochemical, and protein interaction networks. Using this model, Pujana and coworkers successfully identified Hyaluronan Mediated Motility Receptor (*HMMR*), a hub of the integrated network, as a novel high risk associated locus [31]. The gene regulatory network for breast cancer has also been constructed [106]. Taylor and colleagues merged spatial gene expression information with the protein interaction network to highlight the interactions that are active in specific tissues, where the interacting partners are also co-expressed [107]. This work also revealed the loss of key interactions between the network hubs, such as *BRCA1* and their binding partners, in patients who died of breast cancer due to mis-regulation of the partner proteins. In contrast, the expression of hubs and their partners were strongly correlated in surviving patients. The complexity of the disease network is not only restricted to the gene–gene and gene-drug interactions, but also hinges upon the interactions between disease/drug and the host (i.e. genetic background of the patients), as we discuss in the next section.

## From individual network to personalized medicine

**Fig. 4:**
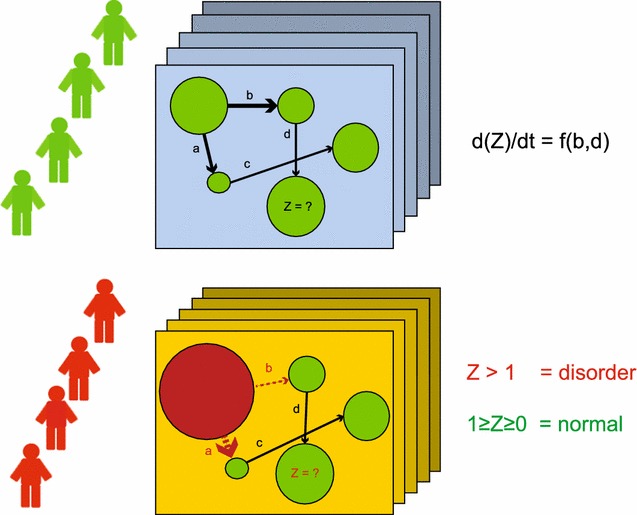
Healthy (*top panel*) and diseased (*bottom panel*) individual networks. Healthy individuals might show slight variations in their individual networks, which also differ over time. However, diseased networks are expected to show greater disparity than that between healthy individuals. In the example shown, the network component *Z* is controlled by its upstream components through the interactions of *b* and *d* (the molecule Z is a function of *b* and *d*). If the expression of *Z* is greater than a defined limit (e.g. 1 in this case), morbidity can be predicted (d(Z)/dt: change of expression level of molecule Z over time)

As we are approaching the so-called personalized and precision medicine era, where does network biology fit in the picture? Figure 4 depicts our view on how networks can be an important tool to help clinicians understand the physiological complexity of individual humans, predict possible failure of certain components that may lead to morbidity, and deduce the most suitable preventative and treatment plans for individual patients. Genetic variation between human individuals is estimated to be less than 1% of the human genome, but through sophisticated regulation of genes and other genetic elements, this small amount of genetic variation accounts for much greater differences in terms of our appearance, intellect, and health [108]. On top of genomes, which encode individual sets of gene products (e.g. proteins, mRNA), individual networks represent the unique interplay between different components in each patient. Understanding the extent of variations between individual networks may allow clinicians to statistically and quantitatively distinguish normal variations in healthy individuals (Fig. 4, upper panel) from critical perturbations that lead to diseases and disorders (Fig. 4, lower panel). Network biology enables researchers to assess multiple components that do not show distinguishable differences between healthy individuals and those with cancers, but are collectively dysfunctional in cancers. A sub-network in which overall activity can be discriminated between patients versus controls has been shown to be a more reproducible prognostic marker of diseases than individual genes in the sub-network, which are not significantly differentially expressed [109, 110].

Single nucleotide polymorphisms (SNPs) and other genetic variations add another dimension of disease-host interaction to disease networks. SNPs can provide clinicians with a good indication on how likely an individual might be to develop certain genetic diseases, assuming that all genetic elements associated with diseases are eventually identified. In addition, networks of individuals can, in part, aid pharmacogenomic progress by explaining why the efficacy and toxicity profiles for the same drug may differ in each patient. For instance, tamoxifen is metabolized by *CYP2D6* and variations in this gene among individuals may affect the response to the drug [111].

No matter how comprehensive, a genetic map cannot capture environmental factors (e.g. lifestyle, contact with pathogens) that heavily influence biochemical stages. Thus, outcomes for the interplay between genetics and environment may be absent in the analysis. Having a network that combines both the genetic variations and measurable biochemical outcomes, such as gene expression, should assist in turning conceptual ideas into more quantitative models, which in turn would enhance the accuracy of prognosis and predictions of disease progression in each patient (as demonstrated in Fig. 4). Such a complete individual network may not be possible in the near future; however, we start to see that the integration of genetic variations and biochemical outcomes (gene expression and protein interaction profiles) has utility in helping identify new disease-associated marker genes [110, 112, 113].

Thanks to considerable effort and resources the community has put into developing computational tools for biological network analysis, we are now well-equipped with a range of user-friendly software that can be employed to handle, visualize, and analyze large-scale datasets. Importantly, the tools that will be particularly useful for translational medical research need to be able to combine multiple layer datasets (e.g. genomics, transcriptomics, proteomics, and metabolomics) and/or heterogeneous datasets (e.g. from different platforms or formats) [3]. The most commonly known network analysis tools currently available are Cytoscape [114], NAViGaTOR [115], VisANT [116], CellDesigner [117], and the commercial software Ingenuity IPA (Ingenuity Systems Inc., Redwood City, CA). More recently introduced tools include NaviCell, which has been developed for online network visualization and curation [118], and BNOmics [119], which can be used for inference and visualization of Bayesian networks of large heterogeneous data. Comprehensive guides to network biology tools, as well as detailed discussion on their key features and functionality can be found in earlier review articles [3, 120].

## Conclusions

Network biology provides an opportunity to image a clear global picture of drug-disease-host interactions and the biological complexity of diseases more easily from an unprecedented top-down vantage. This will allow a better understanding of the relationships between multiple genes and other biological entities, as well as identify the missing links in our knowledge. These strategies are required to fully grasp the intricacies of diseases, which cannot be obtained by studying an individual or a smaller set of genes. The complexity of the therapeutic networks is ever-growing, and many new nodes are being discovered every day. In the future, some of these nodes may become new hubs for targeted therapy.

## Abbreviations

ER: estrogen receptor
FDA: Food and Drug Administration
GRN: gene regulatory network
HER2: human epidermal growth factor receptor 2
HMMR: hyaluronan mediated motility receptor
NGS: next-generation sequencing
PR: progesterone receptor
SNP: single nucleotide polymorphisms
